# Association Between Pre-Pregnancy Body Mass Index and Maternal and Neonatal Outcomes of Singleton Pregnancies After Assisted Reproductive Technology

**DOI:** 10.3389/fendo.2021.825336

**Published:** 2022-01-13

**Authors:** Hanxiang Sun, Yang Liu, Shijia Huang, Xiaosong Liu, Guohua Li, Qiaoling Du

**Affiliations:** ^1^ Department of Obstetrics, Shanghai First Maternity and Infant Hospital, School of Medicine, Tongji University, Shanghai, China; ^2^ Department of Reproductive Immunology, Shanghai First Maternity and Infant Hospital, School of Medicine, Tongji University, Shanghai, China

**Keywords:** pre-pregnancy obesity, BMI, ART, singleton pregnancy, neonatal

## Abstract

**Objective:**

To study the association between pre-pregnancy body mass index (BMI) and adverse maternal and neonatal outcomes of singleton pregnancies after assisted reproductive technology (ART).

**Methods:**

This hospital-based retrospective cohort study of women with live singleton births through ART in China from January 2015 to August 2020 included 3043 Chinese women. According to the latest BMI classification standard of Asian women, the women included in this study were classified as underweight (BMI <18.5 kg/m^2^), normal (BMI 18.5 to <23 kg/m^2^), overweight (BMI 23 to <27.5 kg/m^2^), and obese (BMI ≥27.5 kg/m^2^). We compared the risk of adverse outcomes of different pre-pregnancy BMI values of women with singleton pregnancies conceived through ART. We used Logistic regression analysis to estimate the associations between pre-pregnancy BMI and adverse perinatal and neonatal outcomes.

**Results:**

Our findings showed that women who were overweight or obese before pregnancy through ART are more likely to have a cesarean section, gestational diabetes mellitus, gestational hypertension, and preeclampsia, regardless of whether confounding factors are adjusted. Moreover, pre-pregnancy obesity was more associated with a higher risk of these adverse outcomes than pre-pregnancy overweight. In addition, neonates from women who had obesity before pregnancy through ART were more likely to have macrosomia; adjusted odds ratios and 95% confidence intervals were 3.004 (1.693-5.330).

**Conclusions:**

Our research showed that women who had pre-pregnancy overweight or obesity with singleton pregnancies through ART were more likely to have a cesarean section, gestational diabetes mellitus, gestational hypertension, and preeclampsia. Moreover, neonates from women who had obesity before pregnancy were more likely to have macrosomia.

## Introduction

Studies have shown that being underweight or overweight before pregnancy has adverse effects on pregnant women and their newborns (1–3). Some studies have shown that women with pre-pregnancy underweight are at risk of premature delivery (4), and their newborns are at risk of low birth weight (LBW) and small for gestational age (5). Women with pre-pregnancy overweight have been reported to be at risk of gestational diabetes, cesarean section, gestational hypertension, and postpartum hemorrhage, etc. (6), with increased risks of macrosomia, premature delivery, and neonatal asphyxia in their infants (2). Although studies in different countries have shown different conclusions, it has been established that abnormal weight before pregnancy is related to poor pregnancy outcomes.

Since assisted reproductive technology (ART) has been widely used, it is possible that adverse effects have been studied. Currently, many studies have reported that pregnant women who conceived through ART experience more perinatal complications, and their newborns may have a greater risk of adverse outcomes. A study in Japan showed that pregnancies conceived through ART are prone to a variety of adverse complications, such as premature delivery, placenta previa, placental adhesion, placental abruption, gestational hypertension, etc. (7). A study in Italy reported similar findings among this population (8). Other studies also showed that, even without complications during pregnancy, there was an increased risk of placental adhesion and postpartum hemorrhage during delivery at term (9). Some studies reported that newborns conceived through ART are at a greater risk of premature delivery and LBW (10). However, a prospective study in the United States showed that growth and development in offspring conceived through ART and those conceived naturally were similar, without significant difference (11).

Many studies have explored the relationship of pre-pregnancy weight with live birth rate, pregnancy rate, and abortion rate after ART treatment (12–15). A meta-analysis showed that the clinical pregnancy rate in women with pre-pregnancy underweight was lower than that in women with normal weight before pregnancy, but there was no significant difference between live birth rate and abortion rate (13). A 10-year cohort study in China showed that obese pregnant women receiving ART treatment were at risk of early abortion (14). Some studies also believe that pre-pregnancy weight influences neonatal outcomes in women who conceived through ART. A retrospective cohort study in China showed that maternal overweight and obesity before pregnancy were associated with higher risks of premature delivery, macrosomia, and large for gestational age (LGA) in singleton births conceived through ART. The study also found that the association between pre-pregnancy weight and adverse outcomes was influenced by the time of embryo transfer (fresh/frozen embryo transfer) (16). A study in Slovenia also showed an increased risk of premature delivery in women who had obesity before conceiving through ART (17). A cohort study in Canada found that pregnant women who had pre-pregnancy overweight or obesity had an increased risk of pre-eclampsia, compared with women with normal weight, and that *in vitro* fertilization (IVF) further aggravated the risk of pre-eclampsia, indicating the superposition effect of excessive BMI increase and IVF on the risk of pre-eclampsia (18). Currently, only a few studies have combined ART with pre-pregnancy BMI to study their associations with perinatal complications and neonatal outcomes. In the meantime, there was a lack of research data for Asian populations. Therefore, our study combines two factors—ART and pre-pregnancy BMI—simultaneously to explore the maternal and neonatal outcomes in women who had singleton pregnancies through ART in Shanghai, China.

## Materials and Methods

This retrospective cohort study included women who conceived through ART and delivered to live-born singleton infants at Shanghai First Maternity and Infant Hospital from January 2015 to August 2020. In this study, ART pregnancy refers to pregnancy obtained through IVF or intracytoplasmic sperm injection (ICSI). We reviewed their basic information, including maternal age, gestational age, parity, mode of delivery, premature birth history, rate of gestational weight gain (GWG), and birth year of newborns. The data were obtained from the electronic medical record system of the Shanghai First Maternity and Infant Hospital. This study was approved by the Ethics Committee of the Shanghai First Maternal and Infant Hospital, affiliated with Tongji University School of Medicine. Written informed consent for participation was not required for this study in accordance with the national legislation and the institutional requirements.

### Population

**Figure 1:**
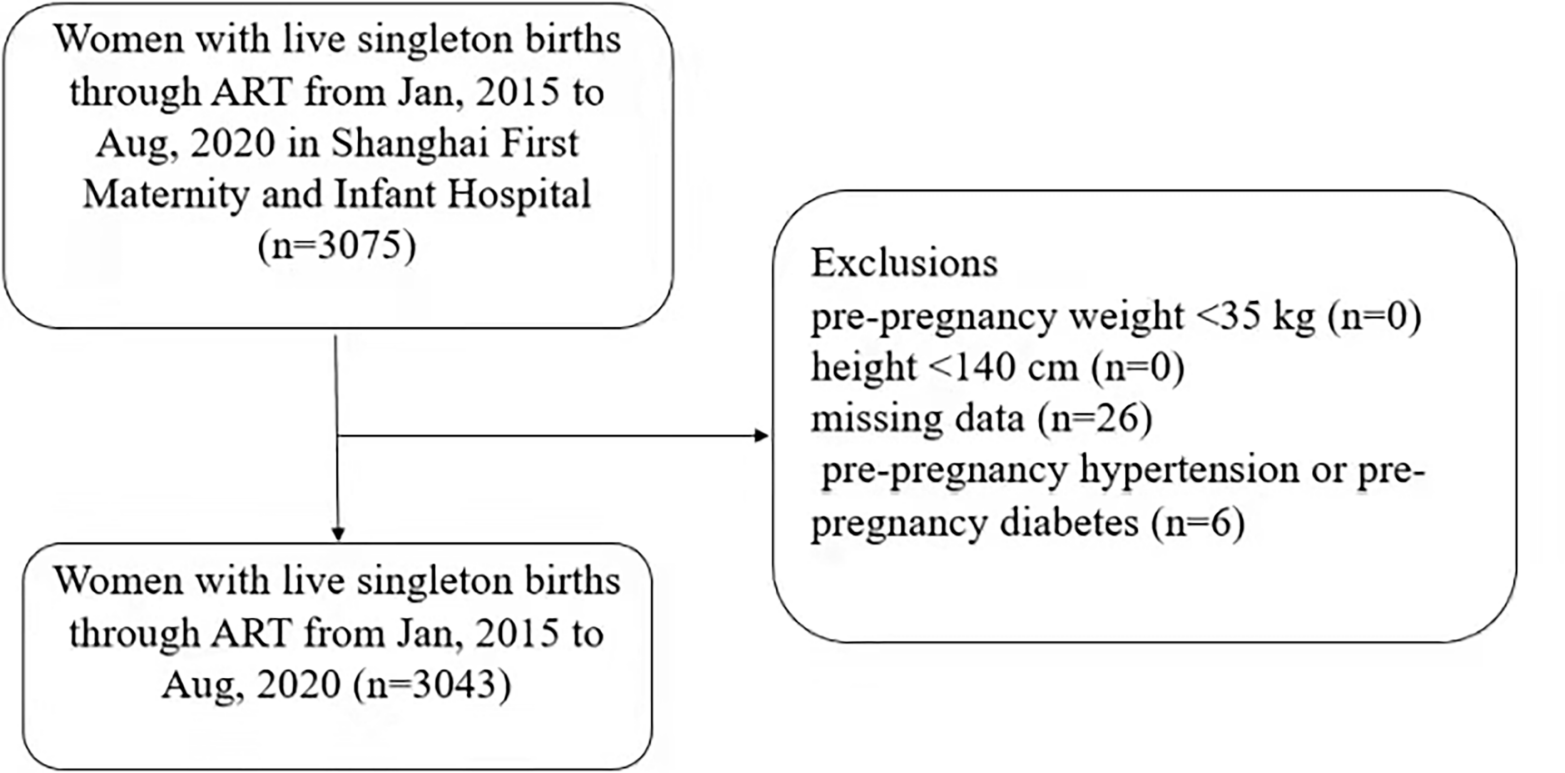
Flow chart concerning the study population.

All women with live-born singleton infants conceived through ART in the information system of Shanghai First Maternity and Infant Hospital from January 2015 to August 2020 were retrospectively selected. Records were deleted from the dataset for the following reasons: pre-pregnancy weight less than 35 kg (n=0), height less than 140 cm (n=0), missing data (n = 26), pre-pregnancy hypertension and diabetes mellitus (n=6). Finally, 3043 patients were included in the study (**Figure 1**).

### Exposure

The primary explanatory variable was maternal pre-pregnancy BMI, defined as the maternal pre-pregnancy weight divided by the square of the height (kilograms/square meters). According to the latest BMI classification standard for Asian women (19), the women included in this study were classified as underweight (BMI <18.5 kg/m^2^), normal (BMI 18.5 to <23 kg/m^2^), overweight (BMI 23 to <27.5 kg/m^2^), or obese (BMI ≥27.5 kg/m^2^). Other studies also believe that it is more appropriate to use BMI classification standards suitable for Asians (20, 21).

### Outcomes

The outcomes of interest are perinatal and neonatal complications. Perinatal complications include gestational hypertension, preeclampsia, gestational diabetes mellitus, premature delivery, premature rupture of membranes, meconium-stained amniotic fluid, polyhydramnios, oligohydramnios, intrahepatic cholestasis of pregnancy, vaginal group B streptococcus infection, thyroid diseases during pregnancy (including hypothyroidism, hyperthyroidism, subclinical hypothyroidism), placenta-related diseases (including placental abruption, placenta previa, placenta adhesion, racket placenta, sail placenta, low-lying placenta, placenta implantation), umbilical cord related abnormalities (including umbilical cord edema, umbilical cord torsion, umbilical cord true knot, umbilical cord spiral, short cord, umbilical cord entanglement, umbilical cord cyst), and postpartum hemorrhage. Newborn outcomes include fetal distress, LBW, macrosomia, newborn sex, and an Apgar score of 1 minute. Pre-pregnancy BMI was calculated according to the height and weight information of pregnant women before pregnancy. The mothers’ pre-pregnancy height and weight were self-reported, and the weight before delivery was measured in the hospital. The gestational age was calculated according to the last menstruation reported during the first antenatal examination. If the fetal size measured by ultrasound is quite different from the gestational age calculated using the last menstruation, the gestational age is calculated according to their earliest ultrasound. Because pregnant women give birth at different times, it would be more accurate for us to use the rate of GWG instead of GWG. The rate of GWG was calculated by subtracting the pre-pregnancy weight from predelivery weight and then dividing it by the number of gestational weeks.

### Statistical Analysis

Continuous variable data are expressed as the mean ± standard deviation (X ± S). The chi-squared test was used to analyze frequency (count) data (%). Statistical significance was set at p <0.05. We used logistic regression analysis to estimate the association between pre-pregnancy BMI and adverse perinatal and neonatal outcomes. The possible confounding factors included maternal age, parity, mode of delivery, the rate of GWG and sex of the fetus. Because maternal age may be an intermediate factor between the pregnant women’s weight and various adverse outcomes, we further stratified the analysis according to maternal age. The association between different pre-pregnancy BMI and adverse outcomes was compared in each layer, and the multiplicative interaction model was used to verify the effect modification. Data analysis was performed using IBM SPSS Statistics for Windows, Version 26.0 (Armonk, NY: IBM Corp).

## Results

In total, 3043 pregnant women receiving ART treatment were included in the study. The proportions of those aged ≤24 years, 25-29 years, 30-34 years, and ≥35 years were 19 (0.62%), 433 (14.23%), 1485 (48.80%), and 1106 (36.35%), respectively. The proportions of those with pre-pregnancy BMI <18.5 kg/m^2^, 18.5-23 kg/m^2^, 23-27.5 kg/m^2^, and ≥27.5 kg/m^2^ were 267 (8.77%), 1765 (58.00%), 834 (27.41%), and 177 (5.82%), respectively. There was no statistically significant difference in the history of premature delivery, primiparity, or multiparity among women across different BMI categories; however, the cesarean section rates in women with pre-pregnancy overweight and obesity were higher at 60.70% and 63.84%, respectively. The vaginal delivery rate of women who were underweight before pregnancy was high (43.07%), and the difference was statistically significant. Compared with the pregnant women with normal weight before pregnancy, the rate of GWG in the overweight and obese groups were 0.33 ± 0.14 and 0.27 ± 0.14 kilograms respectively, and the difference was also statistically significant. The basic characteristics of the women included in this study are shown in **Table 1**.

**Table 1:** Basic characteristics of the study population by pre-pregnancy BMI.

	<18.5 (kg/m^2^)	18.5-<23 (kg/m^2^)	23-<27.5 (kg/m^2^)	≥27.5 (kg/m^2^)
Age, y				
≤24	1 (0.37)	13 (0.74)	3 (0.36)	2 (1.13)
25-29	62 (23.22)	230 (13.03)	113 (13.55)	28 (15.82)
30-34	137 (51.31)	872 (49.41)	381 (45.68)	95 (53.67)
≥35	67 (25.09)	650 (36.83)	337 (40.41)	52 (29.38)
Gestational weeks, day	274.90 ± 11.54	274.28 ± 12.59	272.65 ± 12.96	270.47 ± 16.71
History of preterm delivery, n (%)				
Yes	0 (0)	7 (0.40)	7 (0.84)	1 (0.56)
No	267 (100)	1758 (99.60)	827 (99.16)	176 (99.44)
Parity, n (%)				
Nulliparous	255 (95.51)	1651 (93.54)	764 (91.61)	168 (94.92)
Multiparous	12 (4.49)	114 (6.46)	70 (8.39)	9 (5.08)
Mode of delivery, n (%)				
Vaginal delivery	152 (56.93)	833 (47.20)	333 (39.93)	64 (36.16)
Caesarean section	115 (43.07)*	932 (52.80)	501 (60.07)*	113 (63.84)*
Rate of gestational weight gain, kilogram/week	0.38 ± 0.12	0.36 ± 0.12	0.33 ± 0.14*	0.27 ± 0.14*
Year of delivery				
2015	29 (10.86)	157 (8.90)	81 (9.71)	21 (11.86)
2016	47 (17.60)	284 (16.09)	130 (15.59)	27 (15.25)
2017	45 (16.85)	313 (17.73)	138 (16.55)	28 (15.82)
2018	41 (15.36)	292 (16.54)	153 (18.35)	26 (14.69)
2019	62 (23.22)	408 (23.12)	181 (21.70)	43 (24.29)
2020	43 (16.10)	311 (17.62)	151 (18.11)	32 (18.08)

* P < 0.05.

In the analysis of the relationship between pre-pregnancy BMI and different perinatal complications, we found that the risk of gestational diabetes mellitus, gestational hypertension and preeclampsia in women who had pre-pregnancy overweight and obesity was significantly higher than that in women with normal BMI, and the difference was statistically significant (P <0.05). Regarding other complications, such as premature delivery, premature rupture of membranes, hydramnios, oligohydramnios, intrahepatic cholestasis of pregnancy, positive vaginal group B streptococcus infection, thyroid diseases during pregnancy, placenta-related diseases, umbilical cord-related abnormalities, and postpartum hemorrhage, no statistical difference was found (**Table 2**).

**Table 2:** Associations between pre-pregnancy BMI and perinatal complications in women who conceived singleton pregnancies through ART.

	<18.5 (kg/m^2^)	18.5-<23 (kg/m^2^)	23-<27.5 (kg/m^2^)	≥27.5 (kg/m^2^)
Gestational hypertension and preeclampsia	15 (5.62)	121 (6.86)	124 (14.87)*	39 (22.03)*
Gestational diabetes mellitus	33 (12.36)	285 (16.15)	223 (26.74)*	62 (35.03)*
Premature birth	14 (5.24)	85 (4.82)	42 (5.04)	11 (6.21)
Premature rupture of membranes	46 (17.23)	231 (13.09)	125 (14.99)	27 (15.25)
Polyhydramnios	1 (0.37)	24 (1.36)	11 (1.32)	1 (0.56)
Oligohydramnios	8 (3.00)	44 (2.49)	16 (1.92)	4 (2.26)
Intrahepatic cholestasis of pregnancy	4 (1.50)	22 (1.25)	9 (1.08)	0 (0)
Group B Streptococcus infection	1 (0.37)	17 (0.96)	14 (1.68)	1 (0.56)
Thyroid disease during pregnancy	24 (8.99)	206 (11.67)	94 (11.27)	14 (7.91)
Placental related diseases	36 (13.48)	241 (13.65)	118 (14.15)	20 (11.30)
Umbilical cord related abnormality	5 (1.87)	27 (1.53)	21 (2.52)	5 (2.82)
Meconium-stained amniotic fluid	41 (15.36)	291 (16.49)	121 (14.51)	24 (13.56)
Postpartum hemorrhage	3 (1.12)	6 (0.34)	3 (0.36)	2 (1.13)

* P < 0.05.

In the analysis of the relationship between pre-pregnancy BMI and adverse outcomes in singleton newborns conceived through ART, we found that, compared with pregnant women with normal BMI (5.43%), women who had pre-pregnancy overweight (7.58%) and obesity (13.33%) were more likely to have infants with macrosomia, while women with pre-pregnancy underweight were less likely to have infants with macrosomia (1.72%). Moreover, we also found that the risk of LBW in women with pre-pregnancy obesity was higher than that in pregnant women with normal weight (4.55% vs. 8.67%), and the differences were statistically significant. Regarding other complications, such as fetal distress, gender of newborn, and Apgar score at one minute ≤ 7, no statistical difference was found (**Table 3**).

**Table 3:** Associations between pre-pregnancy BMI and outcomes of singleton newborns after ART.

	<18.5 (kg/m^2^)	18.5-<23 (kg/m^2^)	23-<27.5 (kg/m^2^)	≥27.5 (kg/m^2^)
Fetal distress	12 (4.49)	100 (5.67)	41 (4.92)	12 (6.78)
Gender of newborn	125 (46.82)	965 (54.67)	422 (50.60)	105 (59.32)
^a^Low birth weight	10 (4.29)	73 (4.55)	37 (4.92)	13 (8.67)*
^a^Fetal macrosomia	4 (1.72)*	87 (5.43)	57 (7.58)*	20 (13.33)*
^a^Apgar score at one minute ≤ 7	1 (0.43)	22 (1.37)	7 (0.93)	5 (3.33)

* P < 0.05.

a Due to the partial missing data on neonatal outcomes, 2738 newborns were finally included in the study.

Regarding the perinatal outcome of singleton pregnancy following ART, regardless of whether confounding factors were, the incidence of cesarean section, gestational diabetes mellitus, gestational hypertension and preeclampsia was higher in women with pre-pregnancy overweight and obesity. Regarding LBW and macrosomia, after adjusting for maternal age, primipara or multipara, mode of delivery, the rate of GWG and newborn sex, it was found that women with pre-pregnancy obesity were more likely to have infants with macrosomia, and the adjusted odds ratios (ORs) and 95% confidence interval (CI) were 3.004 (1.693-5.330) (**Table 4**).

**Table 4:** Crude and adjusted OR (95% CI) for the associations between pre-pregnancy BMI and unfavorable outcomes.

	<18.5 (kg/m^2^)	18.5-<23 (kg/m^2^)	23-<27.5 (kg/m^2^)	≥27.5 (kg/m^2^)
^a^Caesarean section Crude OR	0.676 (0.522-0.877)	1 (Reference)	1.345 (1.138-1.589)	1.578 (1.145-2.174)
Adjusted OR	0.699 (0.519-0.943)	1 (Reference)	1.463 (1.195-1.792)	2.233 (1.465-3.405)
^b^Gestational hypertension and preeclampsia Crude OR	0.809 (0.465-1.405)	1 (Reference)	2.373 (1.820-3.094)	3.840 (2.573-5.731)
Adjusted OR	0.728 (0.390-1.360)	1 (Reference)	2.289 (1.709-3.066)	4.365 (2.775-6.866)
^b^Gestational diabetes mellitus Crude OR	0.732 (0.498-1.077)	1 (Reference)	1.895 (1.553-2.313)	2.800 (2.005-3.909)
Adjusted OR	0.882 (0.570-1.365)	1 (Reference)	1.633 (1.295-2.059)	2.004 (1.336-3.006)
^c^Low birth weight Crude OR	0.940 (0.478-1.847)	1 (Reference)	1.085 (0.723-1.627)	1.989 (1.075-3.680)
Adjusted OR	0.798 (0.374-1.704)	1 (Reference)	0.951 (0.612-1.476)	1.338 (0.633-2.826)
^c^Fetal macrosomia Crude OR	0.304 (0.111-0.837)	1 (Reference)	1.429 (1.011-2.020)	2.681 (1.597-4.500)
Adjusted OR	0.312 (0.112-0.872)	1 (Reference)	1.403 (0.964-2.043)	3.004 (1.693-5.330)

a Adjusted for maternal age, parity (nulliparous, multiparous), and rate of GWG.

b Adjusted for maternal age, parity (nulliparous, multiparous), mode of delivery (vaginal delivery, caesarean section) and rate of GWG.

c Adjusted for maternal age, parity (nulliparous, multiparous), mode of delivery (vaginal delivery, caesarean section), sex of newborn and rate of GWG.

We stratified the pregnant women according to their age and analyzed the risk of adverse outcomes among the women and their infants by age and pre-pregnancy BMI. Because there were only 19 pregnant women aged ≤24 years, we did not study their adverse outcomes and those of their newborns. Finally, we found that women with pre-pregnancy obesity (BMI ≥27.5) were more likely to have gestational hypertension and preeclampsia, regardless of their age. We also found that those with pre-pregnancy overweight and obesity aged ≥35 years were more likely to have infants with macrosomia (**Table 5**). In terms of effect modification, we estimated the association between different pre-pregnancy BMI and adverse outcomes according to the age, and added cross-product of age and pre-pregnancy BMI into the model. We found that in the analysis of cesarean section, gestational diabetes mellitus, LBW, macrosomia, gestational hypertension and preeclampsia, the P values of the interaction between pre-pregnancy BMI and maternal age were 0.735, 0.694, 0.207, 0.526 and 0.329, respectively, suggesting that there was no effect modification between pre-pregnancy BMI and age.

**Table 5:** Adjusted OR (95% CI) for the associations between pre-pregnancy BMI and unfavorable outcomes by maternal age.

		<18.5 (kg/m^2^)	18.5-<23 (kg/m^2^)	23-<27.5 (kg/m^2^)	≥27.5 (kg/m^2^)
Maternal age, y					
25-29	^a^Caesarean section	0.819 (0.437-1.535)	1 (Reference)	1.295 (0.766-2.189)	1.808 (0.613-5.332)
	^b^Gestational hypertension and preeclampsia	1.706 (0.528-5.511)	1 (Reference)	2.741 (1.094-6.866)	9.224 (2.321-36.651)
	^b^Gestational diabetes mellitus	0.980 (0.389-2.470)	1 (Reference)	1.325 (0.638-2.754)	1.503 (0.436-5.180)
	^c^Low birth weight	1.148 (0.294-4.486)	1 (Reference)	1.679 (0.592-4.761)	0.887 (0.089-8.879)
	^c^Fetal macrosomia	1.031 (0.265-4.008)	1 (Reference)	1.661 (0.597-4.624)	5.526 (1.144-26.700)
30-34	^a^Caesarean section	0.668 (0.437-1.022)	1 (Reference)	1.528 (1.146-2.039)	3.638 (1.938-6.828)
	^b^Gestational hypertension and preeclampsia	0.459 (0.159-1.323)	1 (Reference)	2.582 (1.684-3.958)	3.633 (1.907-6.918)
	^b^Gestational diabetes mellitus	0.850 (0.445-1.625)	1 (Reference)	1.569 (1.107-2.225)	2.386 (1.362-4.179)
	^c^Low birth weight	0.879 (0.329-2.347)	1 (Reference)	0.859 (0.458-1.611)	1.244 (0.458-3.378)
	^c^Fetal macrosomia	0.139 (0.019-1.033)	1 (Reference)	0.893 (0.502-1.588)	2.245 (1.064-4.736)
≥35	^a^Caesarean section	0.657 (0.356-1.215)	1 (Reference)	1.437 (1.010-2.046)	1.757 (0.741-4.164)
	^b^Gestational hypertension and preeclampsia	0.797 (0.236-2.685)	1 (Reference)	2.059 (1.282-3.308)	3.237 (1.347-7.780)
	^b^Gestational diabetes mellitus	0.725 (0.314-1.676)	1 (Reference)	1.806 (1.274-2.559)	2.271 (1.102-4.680)
	^c^Low birth weight	/	1 (Reference)	0.771 (0.347-1.713)	1.360 (0.354-5.227)
	^c^Fetal macrosomia	/	1 (Reference)	2.167 (1.175-3.997)	3.801 (1.171-12.344)

a Adjusted for maternal age, parity (nulliparous, multiparous), and rate of GWG.

b Adjusted for maternal age, parity (nulliparous, multiparous), mode of delivery (vaginal delivery, caesarean section) and rate of GWG.

c Adjusted for maternal age, parity (nulliparous, multiparous), mode of delivery (vaginal delivery, caesarean section), sex of newborn and rate of GWG.

## Discussion

In this cohort study, 27.41% and 5.82% of women who had singleton pregnancies following ART were overweight and obese, respectively. Our research results showed that women with pre-pregnancy overweight or obesity were more likely to have a cesarean section (adjusted OR 1.463, 95% CI: 1.195–1.792; adjusted OR 2.233, 95% CI: 1.465–3.405), gestational diabetes mellitus (adjusted OR 1.633, 95% CI: 1.295–2.059; adjusted OR 2.004, 95% CI: 1.336–3.006), gestational hypertension, and preeclampsia (adjusted OR 2.289, 95% CI: 1.709–3.066; adjusted OR 4.365, 95% CI: 2.775–6.866). Moreover, those with pre-pregnancy obesity were more likely to give birth to infants with macrosomia in three different age groups; the ORs and 95% CIs were 5.526(1.144-26.700), 2.245(1.064-4.736), and 3.801 (1.171-12.344), respectively, after adjusting for confounding factors.

At present, only a few studies have been carried out on the relationship between maternal pre-pregnancy BMI and perinatal and neonatal outcomes in singleton pregnancies following ART in China. The results of this study show that women with overweight or obesity before conceiving singleton pregnancies through ART were more likely to have a cesarean section, gestational diabetes mellitus, gestational hypertension and preeclampsia. In addition, women with pre-pregnancy obesity were more likely to give birth to infants with macrosomia. A study in China also showed that obesity was associated with an increased risk of macrosomia in ART singleton pregnancies (22). Other studies have shown increased risks of gestational hypertension, preeclampsia, gestational diabetes, and cesarean section in overweight or obese women who conceived through IVF (23–25), which is consistent with our research results. In addition, some studies reported that ART is related to complications such as intrahepatic cholestasis of pregnancy and placental abruption (10). With regard to the outcomes of newborns, some studies have reported that newborns who were conceived through ART were more likely to have macrosomia, especially frozen embryo transfer (26, 27). A previous meta-analysis showed that having a high pre-pregnancy BMI value was more likely to cause macrosomia (2). Our study combines two factors—ART and pre-pregnancy BMI—simultaneously, and showed that women with obesity before conceiving singleton pregnancies through ART were more likely to give birth to infants with macrosomia, which is consistent with findings from previous studies.

Previous studies have shown that maternal age is closely related to adverse pregnancy outcomes, including gestational diabetes mellitus, preeclampsia, premature delivery, placenta previa, LBW, as well as live birth rate (28, 29). A study in Spain also showed that maternal age was associated with a high risk of cesarean section, placenta previa, and gestational diabetes (30). In this study, a stratified analysis of the mothers’ age revealed that women with pre-pregnancy obesity aged ≥35 years were more likely to give birth to infants with macrosomia. It was also found that, regardless of age, women who were pre-pregnancy obesity were at risk of gestational hypertension and preeclampsia. The results suggest that advanced female age is associated with adverse perinatal and neonatal outcomes. In the multiplicative interaction model to verify the effect modification, the P value of the interaction was >0.05, suggesting that there was no effect modification between pre-pregnancy BMI and age.

The physiological mechanism of the effect of pre-pregnancy overweight or obesity on the adverse outcomes of singleton pregnancies following ART is unclear. Obesity can affect the reproductive function of women. A study in the United States showed that, when using autologous oocytes, the higher the BMI of women, the higher the failure rate of intrauterine pregnancy; however, when using donor oocytes, there was no difference in intrauterine pregnancy rate (31), indicating that obesity has adverse effects on oocytes, which also explains why many women with obesity can only get pregnant through ART. One of the most important characteristics of obesity is insulin resistance and hyperinsulinemia. Studies have shown that the systemic inflammatory state and insulin resistance of obese patients are related to the pathogenesis of preeclampsia and gestational diabetes mellitus (32, 33). Fredrik Ahlsson et al. reported that macrosomia is related to the degree of insulin resistance and maternal fat mass; therefore, women with pre-pregnancy obesity are more likely to give birth to infants with macrosomia (34). The cesarean section rate in obese women was higher than that in women with normal weight. Some studies have suggested that the force and frequency of uterine myometrium traction in obese women are smaller; therefore, the incidence of cesarean section is higher (35). The increase in cesarean section rate in pregnancies conceived through ART compared with naturally conceived pregnancies may be due to the high rate of perinatal complications in the former and because the newborns conceived through ART are considered “precious babies”; hence, pregnant women and their families believe that delivery through cesarean section may be smoother. According to a Belgian study, it is an important non-medical factor that the doctors agreed that women who conceived through ART should undergo a cesarean section delivery (36). Studies have suggested the complications in ART conceived singleton pregnancies may be related to drug use (such as hormones) and the ART procedure. Further studies should be carried out regarding the ovarian stimulation regimen, endometrial status of patients, embryo quality, embryo culture time, embryo cryopreservation, and the mechanism of embryo epigenetic modification (37).

The advantages of our study are as follows: the research content is more comprehensive than that of previous studies. Second, the conclusions of our study have a certain guiding role for clinical practice, that is, it is important for women with obesity to reduce weight appropriately before undergoing ART treatment. Moreover, we used the BMI classification for Asians in this study, which is more appropriate for Chinese populations. Our study also had some limitations. First, this was a retrospective study. The pregnant women’s height and pre-pregnancy weight were self-reported, which may have led to a slight overestimation or underestimation of the risks associated with the two measures. Second, we did not consider the causes of infertility in women who conceived through ART to exclude the impact of infertility causes in this study. Third, when stratified by age, the proportion of women aged ≤24 years old was small; therefore, the relationship between this age group and different perinatal outcomes and neonatal outcomes was not studied. In addition, we did not classify ART to further investigate the impact of different types of ART on adverse outcomes, such as fresh embryo transfer or frozen embryo transfer, because some studies think that frozen embryo transfer may increase the risk of macrosomia and large for gestational age (26, 27). 

## Conclusion

In summary, our results suggested that women who had overweight or obesity before conceiving singleton pregnancies through ART were more likely to have a cesarean section, gestational diabetes, gestational hypertension, and preeclampsia. Pregnant women with pre-pregnancy obesity were more likely to give birth to infants with macrosomia. Therefore, it is important to educate women about weight loss before ART.

## Data Availability Statement

The raw data supporting the conclusions of this article will be made available by the authors, without undue reservation.

## Author Contributions

HS and QD drafted the manuscript, analysed and interpreted the data. YL, GL, SH, and XL researched data, conducted statistical analysis, critically revised the manuscript of important content. All authors were involved in writing of the paper and had final approval of the submitted and published versions.

## Funding

This work was supported by Shanghai Science and Technology Commission (grant No. 20Y11907900) and Pudong Municipal Health Commission (grant No. PW2019D-9).

## Conflict of Interest

The authors declare that the research was conducted in the absence of any commercial or financial relationships that could be construed as a potential conflict of interest.

## Publisher’s Note

All claims expressed in this article are solely those of the authors and do not necessarily represent those of their affiliated organizations, or those of the publisher, the editors and the reviewers. Any product that may be evaluated in this article, or claim that may be made by its manufacturer, is not guaranteed or endorsed by the publisher.
